# Genome-Wide Estrogen Receptor Activity in Breast Cancer

**DOI:** 10.1210/endocr/bqaa224

**Published:** 2020-12-07

**Authors:** Anca M Farcas, Sankari Nagarajan, Sabina Cosulich, Jason S Carroll

**Affiliations:** 1 Bioscience, Oncology R&D, AstraZeneca, Cambridge, UK; 2 CRUK Cambridge Institute, University of Cambridge, Cambridge, UK; 3 Division of Molecular and Cellular Function, School of Biological Sciences, Faculty of Biology, Medicine and Health, The University of Manchester, Manchester, UK; 4 Projects Group, Oncology R&D, AstraZeneca, Cambridge, UK

**Keywords:** Breast cancer, estrogen receptor, chromatin, enhancers, cofactors

## Abstract

The largest subtype of breast cancer is characterized by the expression and activity of the estrogen receptor alpha (ERalpha/ER). Although several effective therapies have significantly improved survival, the adaptability of cancer cells means that patients frequently stop responding or develop resistance to endocrine treatment. ER does not function in isolation and multiple associating factors have been reported to play a role in regulating the estrogen-driven transcriptional program. This review focuses on the dynamic interplay between some of these factors which co-occupy ER-bound regulatory elements, their contribution to estrogen signaling, and their possible therapeutic applications. Furthermore, the review illustrates how some ER association partners can influence and reprogram the genomic distribution of the estrogen receptor. As this dynamic ER activity enables cancer cell adaptability and impacts the clinical outcome, defining how this plasticity is determined is fundamental to our understanding of the mechanisms of disease progression.

## “We Are Not There Yet”—Breast Cancer Remains a Clinical Challenge

Despite substantial achievements in our understanding of breast cancer and the advancement in treatment options, it currently represents the most common cancer in women worldwide (approximately 2 million new cases diagnosed in 2018 alone) (1), with estimates indicating that 1 in 8 women will develop breast cancer in their lifetime (American Cancer Society Facts & Figures 2020 & DevCan/National Cancer Institute). Although each subtype is highly heterogeneous (2-5), hormone receptor positive (HR+) tumors comprise up to 70% to 80% of breast cancer cases and are driven by the estrogen receptor alpha (ERα, henceforth ER) transcription factor. The role, if any, of ER beta (ERβ) in breast cancer is unclear and will not be covered in this review. Indeed, treatment options broadly termed “endocrine therapy” targeting either estrogen production (ie, aromatase inhibitors such as anastrozole, exemestane, or letrozole) or the hormone receptor itself such as selective estrogen receptor modulators (SERMs; ie, tamoxifen) and selective estrogen receptor degraders (SERDs; ie, fulvestrant) have proven highly effective, contributing to the 39% decrease in breast cancer mortality rates observed in the UK between 1971-1973 and 2015-2017 (Cancer Research UK).

More recent success obtained in locally advanced or metastatic HR+ breast cancer using CDK4/6 inhibitors in combination with endocrine therapy has changed treatment practice (6-9). Furthermore, the development of orally bioavailable SERDs (10-13) or emerging technologies such as PROteolysis TArgeting Chimeras (PROTACs) (14-16) have added value and potential to our future portfolio of treatment options. Nonetheless, the adaptability of cancer cells means a significant percentage (25%-30%) of early ER+ breast cancer patients either acquire or present with *de novo* resistance and stop responding to standard-of-care treatments (17, 18), inevitably leading to incurable metastatic disease. As endocrine therapy resistance represents the lethal, treatment-refractory form of the disease, there is a need to define the molecular mechanisms and additional players involved in the ER-dependent transcriptional program, with a focus on the proteins that contribute to resistance and might constitute novel therapeutic opportunities.

## Estrogen Receptor Binds DNA at Enhancers, Which Are Distal Regulatory Elements Essential for Activation of Tissue-Specific Transcriptional Programs

Genome-wide chromatin binding profiling revealed that ER (and other nuclear hormone receptors) primarily localize away from transcription start sites to cis-regulatory regions called enhancers (19-21). These relatively short DNA enhancer elements (~ 500-1000 bp in length on average) play an essential role in ensuring a coordinated cell type specific gene expression program during development and differentiation, with increasing evidence to suggest that global enhancer activation is closely linked to cancer development and tumor aneuploidy (22). These regulatory elements contain a dense number of binding sites for multiple transcription factors (TFs), thereby enabling them to act as “integration hubs” and sensors of the cell state. Enhancers have been broadly annotated based on their specific chromatin signature, including binding by the acetyltransferase p300 or occupancy of other key cofactors; the presence of Mixed Lineage Leukemia (MLL) 3/4-directed mono-methylation of H3K4 (H3K4me1); low nucleosomal density; the presence of H3.3 and H2A.Z histone variants contributing to “high mobility” nucleosomes; and depending on transcriptional activation status, p300-mediated acetylation of H3K27 (H3K27ac).

Importantly, enhancers can stimulate promoter activity over long genomic distances, being located anywhere between 1 kb to more than 1 Mb away from the target gene. With up to 400 000 putative enhancers proposed in the mouse and human genomes (23-25), it has proven difficult to assess what proportion are actually functional, and a universal molecular mechanism of how enhancers promote target gene transcription remains elusive. One prevalent model proposed to understand enhancer-promoter “communication” involves loop formation to bring into close proximity these two regulatory elements while physically excluding the “passive” intervening DNA, an interaction that is mediated and stabilized by the insulator protein CCCTC-binding factor (CTCF) and the ring-shaped Cohesin complex (26-30). Interestingly, the distinction between enhancers and promoters is becoming increasingly blurry, with not only sharing chromatin features and regulatory factors both but also functional roles. Promoters displaying bidirectional activity can function as enhancers, while enhancers are able to drive transcription of short, noncoding, and unstable transcripts called enhancer RNAs (eRNAs) or act as alternative tissue-specific promoters (31-36). The exact function, if any, of eRNAs continues to be controversial. Some specific eRNAs have been shown to be functional, with a proposed role in enhancer-promoter loop formation or stabilization, potentially through interactions with subunits of the Cohesin and Mediator complexes (37-41). However, other work suggests that the production or accumulation of eRNAs is not required for ER complex assembly on enhancers or for looping to gene promoters (42) and may instead be a by-product of RNA polymerase II occupancy at active enhancers (43).

The chromatin looping model has been informed by the advent of chromatin conformation capture–based technologies (44-48). Together, these led to the description of genome organization into compartments called topologically associating domains and the detection of extensive long-range interactions occurring within the nuclear space. On this three-dimensional (3D) genome organization scale, estrogen stimulation was shown to induce enhancer-promoter looping (49, 50) and a more global higher-order recompartmentalization of chromatin domains (51-53) in a relatively short amount of time to allow a coordinated transcriptional response. The relevance of these long-range chromatin interactions to breast cancer is demonstrated by work showing that aberrant genomic amplification of ER-occupied enhancers can negatively impact on survival and therapy response through the formation of novel rogue long-range target-promoter interactions (54-56). Furthermore, an altered chromatin organization pattern (51) and differential chromatin interactions both within and between topologically associating domains were found to be associated with endocrine resistance (57), indicating that 3D epigenome dysregulation is a common feature of transformed cells and of treatment-refractory breast cancer.

**Figure 1.**
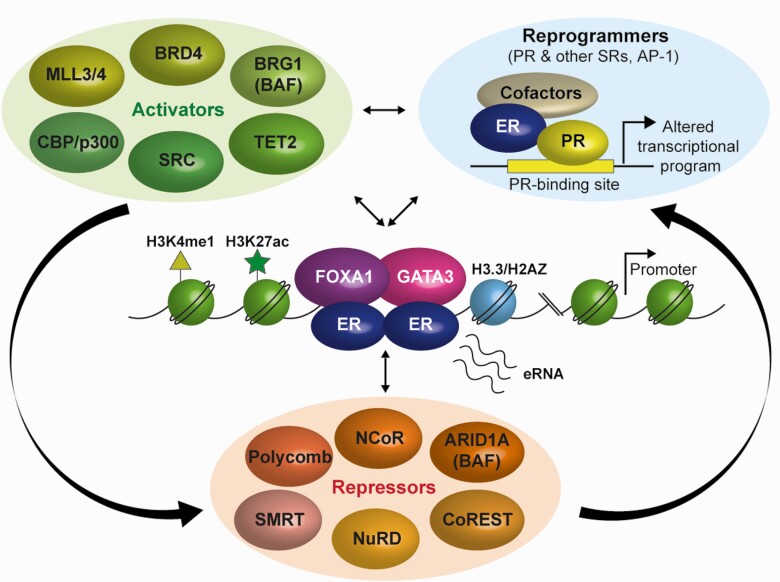
The functionality of ER-occupied enhancers is determined by an interplay between factors with opposing activities. The estrogen receptor largely occupies cis-regulatory elements called enhancers, genomic regions which are characterized by unique chromatin features. These regulatory elements are generally situated at large distances from their target gene promoter. ER binding to enhancers is mediated by FOXA1 and GATA3, factors required for ER to access compacted DNA and ultimately, for estrogen-dependent transcription and cell growth. A number of other proteins such as PR and other steroid receptors or AP-1 transcription factors were also reported to influence ER-driven gene expression and clinical outcome by redirecting (“reprogramming”) ER to novel binding sites. Once bound, the functionality of ER-occupied enhancers relies on the multitude of cofactors with opposing activities (examples depicted are illustrative but not exhaustive), which mediate the response to hormone signaling. Identifying and understanding the contribution of these players to ER-mediated gene regulation holds potential for therapeutic intervention and drugs targeting some of these factors are being actively developed and assessed.

Understanding and defining enhancer function and its role in 3D genome organization continue to represent exciting areas of intense research, with a number of excellent reviews summarizing current knowledge in the field and discussing alternatives to the chromatin looping model (31, 58-65). Here, we specifically focus on factors reported to play a role in ER-regulated enhancer functionality (Fig. 1 & Table 1) and discuss recent developments regarding reprogramming of the ER cistrome (ie, its global chromatin binding profile) and how this influences the transcriptional program and treatment response.

**Table 1.** Examples of Coactivators and Corepressors That Regulate the ER-Driven Transcriptional Program

Factors	General function	Reported ER-specific function	Refs
**Coactivators**			
p300/CBP	Histone acetyltransferase (HAT) mediating histone acetylation, importantly on H3K27, modification found at active gene regulatory elements	Forms core complex with ER and is required for enhancer activity and target gene expression	(66, 67)
SRC1/2/3	P160/nuclear receptor coactivator (NCoA) family. Possesses p300/CBP interaction domain and weak HAT activity	Forms core complex with ER and mediates the interaction between p300 and ER	(68, 69)
BRG1	ATPase subunit in the SWI/SNF chromatin remodeling complex	Directly interacts with ER and is required for efficient ER-driven transcriptional activation	(70)
CARM1	Arginine methyltransferase with activity toward histone and nonhistone substrates	Involved in both estrogen-induced activation and repression of genes; implicated in tamoxifen-resistant/ligand-independent activation of ER	(71, 72)
TET2	Active DNA demethylation activity	Binds active enhancers and facilitates the recruitment and function of ER	(73)
**Corepressors**			
CoREST	Complex with repressor activity and a subunit composition including HDACs, the lysine demethylase KDM1A/LSD1, and REST corepressor (RCOR) proteins	Transcriptional repressor for ligand-activated ER; CoREST subunits identified as required for gene regulation by ER and for ER-mediated cell growth	(74)
NCoR & SMRT	Transcriptional corepressors forming large assemblies which include HDAC activity	Associated with estrogen-suppressed genes and SERM-mediated response	(75, 76)
**ARID1A**	BAF chromatin remodeling complex; reported tumor suppressor gene implicated in various cancers	Recruited during tamoxifen treatment; mediates tamoxifen and fulvestrant response	(77, 78)
**NuRD**	Chromatin remodeling and HDAC complex	Associated with tamoxifen-induced recruitment	(74, 79-88)
**Polycomb group proteins**	Transcriptional repressors of developmental regulatory loci	Diverse links with ER biology reported; BMI1 & EZH2 associated with tamoxifen resistance, while RING1B linked with estrogen-driven gene activation	(89-92)

List of coregulators discussed in the text, with a description of their general function and reported role in ER biology (list is not exhaustive and multiple other factors have been described to play a role in hormone-dependent signaling). The 3 factors we discuss in greater detail are shown in **bold**. References used in the main text relevant to their reported role in ER-bound enhancer functionality are included.

Abbreviations: ARID1A, AT rich interactive domain 1A; ER, estrogen receptor alpha; HAT, histone acetyltransferase; HDAC, histone deacetylase; NCoA, nuclear receptor coactivator; NuRD, nucleosome remodeling and deacetylase; SERM, selective estrogen receptor modulator.

## The Repertoire and Function of ER Targeted Enhancers Depend on the Identity and Activity of its Coregulators

One of the main players in ER biology is represented by the pioneer transcription factor Forkhead Box A1 (FOXA1). The main feature of pioneer factors is their ability to access their binding motifs in closed chromatin, thereby promoting a local chromatin decompaction which enables the binding of canonical TFs and subsequent lineage-specific patterns of active enhancers and unique transcriptional programs. Despite discussions of whether steroid receptors can reprogram a pioneer TF at discrete subsets of genomic loci (93, 94), it is clear that the vast majority of ER binding events are dependent on the action of FOXA1 (95). As ER binding and cell proliferation show a dependency on FOXA1 even in an endocrine-resistant setting (95-97), FOXA1 represents an attractive (yet currently undruggable) therapeutic target in breast cancer, with FOXA1 inhibitors potentially also effective where resistance to ER antagonists has emerged.

Alongside FOXA1, several coactivators are found at ER-bound enhancer elements, including the steroid receptor coactivators SRC1/2/3 (also known as nuclear receptor coactivator NCoA1/2/3) (68, 69), the acetyltransferases p300/CBP (66, 67), the protein arginine methyltransferase CARM1 (71, 72), and the methylcytosine dioxygenase enzyme TET2 (73). Together, coactivators facilitate gene expression through a combination of mechanisms, including modifying the chromatin landscape and recruiting the general transcriptional machinery (98, 99). As disruption of many of these coactivators impairs the estrogen response, they are attractive drug targets and various inhibitors against some of these factors were reported to exhibit anti-proliferative activity in ER+ breast cancer (100-102) or androgen receptor (AR)+ castrate-resistant prostate cancer (103, 104) preclinical models.

While the role of coactivators is better understood, increasing evidence suggests that transcriptional corepressors such as the nucleosome remodeling and deacetylase (NuRD) complex, nuclear receptor corepressor (NCoR), or silencing mediator of retinoic acid and thyroid hormone receptor (SMRT) (75, 76) also occupy active regulatory regions together with coactivators (105, 106). The resolution of population-type experiments such as chromatin immunoprecipitation-sequencing (ChIP-seq) does not reveal whether coactivators and corepressors co-occupy chromatin together at a single locus level or whether this is a dynamic process and these opposing classes of cofactors are mutually exclusive. However, there is evidence to suggest that regulated gene expression depends on a dynamic balance between coactivators and corepressors working antagonistically (107, 108), and that indeed, in some cases, gene expression is impaired upon depletion of a repressor. As an example, histone deacetylase (HDAC) inhibition resulted in impaired *c-fos* and *c-jun* gene induction despite enhanced histone acetylation on those targets, indicating that the active and dynamic turnover between acetylation and deacetylation is required for robust gene expression (109).

Interestingly, recent mass spectrometry–based absolute quantification work revealed that, on average, corepressors are ~ 100 times more abundant than coactivators, meaning that, overall, the nuclear environment is highly repressive (110). This abundance indicates that repressors may play a more global role in gene regulation beyond their recruitment by TFs to mediate locus-specific gene silencing. Several repressive factors and complexes were linked with ER signaling and we will describe 3 such examples (AT rich interactive domain 1A [ARID1A-BAF], NuRD and Polycomb group), selected based on frequency of alterations in ER+ breast cancer (ARID1A); a functional link with ER biology and tamoxifen response (NuRD); and an unexpected role for a transcriptional repressor in the activation of ER-bound enhancers (Polycomb). Although their link with ER biology is better understood in the case of ARID1A-BAF and perhaps less established in the case of NuRD and Polycomb, these examples nonetheless illustrate advances made in our understanding and novel avenues being explored of how repressive complexes can contribute to enhancer functionality in HR+ breast cancer.

### ARID1A-BAF complex

Consistent with their fundamental role in transcriptional control and genome integrity, members of the ATP-dependent chromatin remodeling complexes are frequently mutated at the somatic or germline level across cancer types (111-113). For example, inactivating mutations in the components of the Switch/Sucrose nonfermentable (SWI/SNF) complex (referred to as the BRG1/BRM-associated factor/BAF complex in humans) are collectively found in a quarter of all cancers (114, 115). BAF members are differentially impaired depending on the cancer type, with ER+ breast cancer primarily characterized by aberrations in the AT rich interactive domain 1A (ARID1A/BAF250A) subunit (~ 5% at the primary tumor and ~ 12% in resistant/metastatic setting) (116-118). Ligand-dependent association between ER and the BAF ATPase catalytic subunit BRG1 and the critical role for BRG1 as a coactivator in ER-mediated transcriptional regulation were previously reported (70). However, the role of the ARID1A BAF subunit in hormone-dependent breast cancer was mechanistically underexplored.

Two recent papers reveal that loss of ARID1A promotes endocrine therapy resistance (77, 78). In terms of the precise mechanism by which loss of ARID1A contributes to a poorer clinical outcome, there are discrepancies between the two studies. For example, Xu et al (77) suggest that knockout of ARID1A not only destabilizes the BAF complex binding to chromatin, but it significantly impairs a subset of the cistrome of both ER and FOXA1 at BAF co-occupied loci, with the ultimate result being ER-independent growth and a transition to a more aggressive basal-like transcriptional program. In contrast, Nagarajan et al (78) do not detect any significant change to ER chromatin binding. Instead, they show that ARID1A is part of the ER complex and that it acts as a repressor, with its loss resulting in an altered ARID1A-occupied enhancer landscape characterized by dysfunctional BAF complex occupancy, a decrease in HDAC1 binding and a subsequent increase in enhancer-specific acetylation. The higher acetylation is accompanied by increased recruitment of the histone acetylation reader BRD4, a member of the bromodomain and extra terminal domain (BET) protein family, with the end result of BET-dependent activation of genes normally repressed by tamoxifen in the ARID1A wild-type context. A role for the epigenetic reader BRD4 as a regulator of ER biology and the potential of BET inhibitors in the treatment of various solid tumors was previously established (119-122). This increased dependency on BRD4 was therefore exploited to show that BET inhibition has a significant antiproliferative effect in the ARID1A mutant context.

While the differences between the 2 studies underline the complexity of disentangling the change in the enhancer landscape upon BAF impairment, both papers link ARID1A mutations to changes in ER function and treatment response. These studies also provide a feasible and actionable strategy for the clinic, first as an opportunity to stratify patients based on ARID1A aberrations, with patients harboring mutant ARID1A more likely to relapse on endocrine therapy. Second, these studies highlight the need to explore the vulnerabilities or synthetic lethality opportunities arising from these alterations, such as an increased dependency on BRD4 or the identification of ARID1B as a vulnerability in ARID1A-mutant cancers (123, 124).

### NuRD complex

While defined as a transcriptional repressor, the NuRD complex can also bind accessible regions such as active enhancers and promoters. The cistrome of various NuRD subunits, including the ATPase protein Chromodomain helicase DNA-binding protein 4 (CHD4), largely overlaps with the profile of the BAF catalytic subunit BRG1 in a variety of cell types (125-127). In addition to the mutually exclusive NuRD ATPase subunits CHD3 and CHD4, the complex contains several other proteins including the HDAC1/2 deacetylases, the metastasis-associated proteins MTA1/2/3, the GATA zinc-finger domain containing GATAD2A/2B factors or the histone lysine demethylase 1A KDM1A/LSD1, although the specific composition of proteins appears to be context-dependent (128-130). Taken together, NuRD combines chromatin remodeling and HDAC activity to mediate target gene repression, and its essential role in cell fate specification across a range of developmental contexts has been reported (131, 132).

Multiple links between NuRD and ER biology were proposed (79, 80). For example, NuRD was reported to be recruited by the TWIST transcription factor to repress ER expression and therefore promote loss of response to endocrine therapy and breast cancer progression (81). Furthermore, MTA3 was shown to be an estrogen-dependent NuRD member playing a role in regulating epithelial to mesenchymal transition (82). Despite these links, clear mechanistic insight into the role of NuRD in the ER-driven transcriptional program is missing, potentially complicated by the context-dependent composition of the complex, its involvement in a variety of nuclear processes, and its ability to function with multiple TFs (ie, not ER-specific). The recruitment of NuRD by ER following tamoxifen treatment is well-established and fits into the canonical view of a repressor complex being specifically recruited by a TF to mediate gene silencing in the context of drug-induced repression (83, 84). However, finding the NuRD corepressor complex at active gene regulatory elements is unexpected and needs to be mechanistically explored. One explanation could be that NuRD binding is a consequence of indiscriminate occupancy of accessible regions. Alternatively, this binding profile could suggest NuRD may be required for fine-tuning of gene expression and enhancer functionality, in both ligand-independent and -dependent contexts. Interestingly, CHD4 was recently reported to play an essential role in keeping super-enhancers (ie, clusters of enhancers) open and permissive to the binding of the oncogenic transcription factor PAX3-FOXO1 in rhabdomyosarcoma (133), suggesting this NuRD component may be a promising target for super-enhancer disruption therapies (134).

While missense loss-of-function mutations which affect the ATPase domain of CHD4 are considered a driver in endometrial carcinoma (111, 135, 136), such mutations are not as prevalent in invasive breast cancer. Nonetheless, NuRD subunits are part of the ER interactome in breast cancer cells even in the absence of tamoxifen treatment (83) and multiple recent reports have linked this complex with the GATA family transcription factor trichorhinophalangeal syndrome type I (TRPS1), which is frequently amplified in breast and prostate cancer (74, 85-88). Focusing on hormone-sensitive breast cancer, TRPS1 was proposed to specifically recruit NuRD in order to suppress cell migration and invasion genes, thereby reducing the metastatic potential of breast cancer cells (87). Serandour et al (74) identified the NuRD complex as being essential for ER+ breast cancer cell growth and highlighted an association at the proteomic level between TRPS1 and repressive activities represented by both NuRD and CoREST. While unable to show whether loss of TRPS1 impairs the cistrome of the NuRD ATPase subunit CHD4, the observation that depletion of TRPS1 leads to a redistribution of ER chromatin binding suggests that corepressors such as NuRD may play a broader role as modulators of TF binding potential and enhancer function which warrants further investigation.

### Polycomb repressors at active ER-occupied enhancers

Polycomb group proteins are essential regulators of gene expression, acting as transcriptional repressors of developmental regulatory loci such as the Hox cluster (137-140). Largely divided into 2 main complexes, namely Polycomb repressive complex (PRC) 1 and 2, these epigenetic players and their associated chromatin modifications were reported to primarily occupy promoters of silenced genes (141-143). However, increasing evidence suggests that Polycomb proteins are more broadly distributed and can also bind active regulatory regions in a variety of cell types (144-147). While their precise contribution to gene activity remains unclear (148), some proposed examples include a role in facilitating enhancer-promoter interaction (149, 150) or indirectly influencing RNA polymerase II activity (151).

Surprisingly, the PRC1 E3-ubiquitin ligase RING1B itself was reported to be recruited in an estrogen-dependent manner to active enhancers co-occupied by ER, FOXA1, and another ER-cooperating transcription factor called Grainyhead Like Transcription Factor 2 (GRHL2) (89, 90). At these loci, RING1B was proposed to play a role in regulating the chromatin accessibility and activity of *de novo* (not marked with high levels of H3K27ac prior to estrogen stimulation) enhancers. The observation that chromatin accessibility at estrogen-stimulated genes is dynamic and cyclical in a manner that seems dependent on RING1B raises the possibility that this Polycomb factor plays a role in resetting the ER-occupied enhancer chromatin landscape to allow the cell to be adaptive and responsive to environmental stimuli, although mechanistic insight into this will require additional work. How depletion of RING1B impairs accessibility specifically at these *de novo* enhancers and whether this is a cause or instead a consequence of the reduced ER binding seen following Polycomb knockdown requires further exploration. While the E3 ligase activity of RING1B appears to be important, it remains unclear whether this is due to impaired ubiquitination of its histone target (H2AK119) or loss of activity toward a nonhistone target, perhaps a TF such as ER. Unlike RING1B which seems required for estrogen-induced gene expression, another E3 ubiquitin ligase complex consisting of RNF20/RNF40 was shown to repress estrogen-inducible enhancers in a manner dependent on its activity toward the histone substrate H2B, underlining the complex chromatin landscape characterizing these regulatory elements (152).

In addition to RING1B, other Polycomb group proteins were linked to ER signaling, with both the PRC1 component B lymphoma Mo-MLV insertion region 1 homolog (BMI1) and the PRC2 catalytic subunit Enhancer of zeste homolog 2 (EZH2) reported to contribute to tamoxifen resistance (91, 92). While the role of Polycomb repressors in the functionality of ER-occupied enhancers is far from clear, it is important to note that Polycomb players are routinely identified in unbiased CRISPR-based screens in multiple cancer types as either essential for tumor viability or instead required for treatment response (153-155). These studies underline the importance of cellular context and disease stage when trying to establish the role of these epigenetic regulatory proteins. With the exciting development of clinical-grade PRC2 inhibitors (156-158), targeting Polycomb proteins may represent an opportunity to therapeutically exploit the epigenetic plasticity rendered by these factors in ER+ breast cancer.

## Enhancer Plasticity Equals Tumor Adaptability—Enhancer Reprogramming During Disease Progression Leads to Altered Transcriptional Programs

For a TF such as ER, there are many potential binding sites throughout the genome, which can be attained either directly via its own DNA-binding domain, or indirectly, via recruitment by other factors. However, only a minority of these binding sites are occupied at any given time. The vast repertoire of possibilities enables plasticity in terms of a TF cistrome leading to highly adaptable transcriptional programs. While adaptability allows cells to quickly respond to environmental stimuli, it can also be associated with disease transition, providing a proliferative benefit to a handful of cells leading to unregulated growth. Understanding the constraints on adaptability is an exciting therapeutic avenue, as it may provide an opportunity to define new ways to limit tumor growth. Dissecting the identity and expression levels of ER interaction partners is crucial to building our understanding of how such a dynamic ER cistrome is determined.

The ER cistrome is reprogrammed both during the transition from normal mammary epithelial tissue to primary tumor and also when endocrine resistance develops. Due to a lack of good models and scarcity of material, the role of ER in the early events of tumor development remain poorly explored. However, the chromatin binding profile of ER is different in normal mammary epithelial cells compared with ER+ tumor specimens, a differential cistrome which translates into distinct gene expression patterns (159). It is not clear whether the reprogrammed ER is causative or a consequence of tumorigenesis, but analysis of the differentially expressed genes revealed a concordance between changes in ER binding and a shift to a more pro-oncogenic transcription program. Interestingly, many more ER binding events were identified in tumors when compared with normal mammary epithelial cells. This could be due to changes in ER interaction partners or changes to factors which influence ER binding. For example, both FOXA1 and GATA3 were reported to show higher expression in tumor versus normal tissue (160, 161). Increased ER binding also suggests that the transition to tumorigenesis may be characterized by an overall increase in genome accessibility, thereby exposing more potential TF binding sites.

Multiple studies implicate an altered FOXA1 activity in mediating endocrine resistance and resulting poor clinical outcome (19, 95, 162, 163). ER chromatin distribution is different in primary tumors from patients who responded to treatment compared with patients who relapsed and metastasized, and the FOXA1 transcription factor binding motif is enriched at the novel ER-occupied enhancers in the poor outcome setting (19). While this suggests a primarily FOXA1-driven redistribution of the ER cistrome resulting in a more aggressive transcriptional signature in worse outcome patients, what drives the abnormal FOXA1 profile is not clear. Furthermore, in tamoxifen-resistant ER+ cell lines, hyperactive FOXA1 was reported to drive a pro-metastatic transcriptional program through assembly at and activation of novel enhancer elements (163). Interestingly, not all of these FOXA1/H3K27ac-gained enhancers are co-occupied by ER, indicating that FOXA1 may co-operate with other TFs in mediating endocrine resistance. In agreement, when comparing the ER cistrome between tamoxifen-resistant versus tamoxifen-sensitive cells, Bi et al identified a global redistribution of ER binding, with the Activator protein 1 (AP-1) motif preferentially enriched on the gained enhancers and the GATA3 motif on the lost enhancers (164). This reprogrammed ER cistrome is therefore determined by interactome changes, whereby differential association with distinct TFs can tether ER to their own respective binding sites and drive altered signaling programs. Therefore, manipulating this interplay between ER and its interaction partners could be used to restore tamoxifen sensitivity or prevent the development of endocrine resistance.

Along these lines, recent evidence indicates that activating the progesterone receptor (PR) in ER+, PR+ breast cancer drives a unique transcription program that is associated with a good clinical outcome (165). In particular, upon treatment with native progesterone or the synthetic progestin R5020, a robust association was detected between activated PR and ER, with liganded PR shown to redistribute ER chromatin binding events toward progesterone-response elements, the binding motif for the PR transcription factor. Importantly, these novel ER-occupied enhancers are also characterized by recruitment of the coactivator protein p300, suggesting that the reprogrammed ER cistrome is transcriptionally functional. Analysis of the induced genes showed enrichment for antiproliferative pathways, an effect that was reproduced in tumor explants and xenograft models when estrogen and progesterone were administered in combination. Similarly, multiple reports indicate extensive interplay between other steroid receptors such as the glucocorticoid receptor and the androgen receptor and ER signaling (166-169). Therefore, depending on the identity of the associating partner and their effect on the ER cistrome, this reprogrammed enhancer configuration can be exploited therapeutically. Ultimately, this dynamic ER interactome and cistrome and its association with clinical outcomes underlines our growing understanding that multiple factors contribute to endocrine therapy response in addition to ER itself.

## Manipulating Transcription Factor Dynamics—An Alternative Strategy to Targeting ER?

For an efficient and sustained antiproliferative effect, an approach that considers not just ER in isolation but also its varying nuclear environment and interactome repertoire is likely to be required. For maximum antagonist activity (loss of estrogen-driven gene expression and cell proliferation), drugs which achieve highest ER degradation have been assumed to be best. However, recent work suggests that estrogen receptor degradation may partially be a consequence of ER antagonism (10) and that the multiple novel SERD compounds currently being developed (11, 12, 170-172) differ in their specific abilities to affect the nuclear mobility of the estrogen receptor—with the best antagonist causing the greatest degree of ER physical immobility. This effect on ER nuclear mobility was proposed to be mediated via compound-specific induced changes in the ER interactome. By this logic, potent antagonists would not only achieve an efficient dispersal of coactivators but could potentially also promote an association with factors responsible for reducing ER intranuclear mobility. Interestingly, proteins such as the scaffold attachment factors B1 and B2 (SAFB1/2) were reported to slow down the nuclear mobility of liganded ER by extending its dwell time on the nuclear matrix (173). As this higher retention on the matrix was proposed to lead to impaired ER-driven transcription due to reduced ER binding to chromatin, the role of the nuclear matrix in regulating ER mobility could represent an interesting and novel avenue to explore.

It remains unclear how reduced nuclear mobility is linked to the generation of a transcriptionally inert ER complex on target enhancers and subsequent degradation of the estrogen receptor. However, prior to ER degradation, SERDs induce ER association with chromatin in a manner comparable to estrogen stimulation (10). This raises the question of whether ER is degraded while bound to chromatin or whether ER needs to shuttle off chromatin and be localized in the nuclear space for efficient degradation. Although a mechanism of shuttling remains unclear, the cyclical manner in which ER reportedly binds target cis-regulatory elements suggests the existence of mechanisms for displacement of ER from chromatin.

Binding of ER is highly dynamic, with a residence time in the range of seconds (93, 174). In addition to this fast binding kinetics observed at the individual molecule level, a cyclic chromatin binding pattern at a population level was reported for ER and some of its key cofactors, with a periodicity of occupancy of approximately 90 minutes despite constant estrogen stimulation (175, 176). While these studies focus on a panel of ER targets, which may not necessarily mean this cycling pattern is valid across all estrogen-regulated genes, they do nonetheless show that this binding pattern of the ER complex correlates with cyclic levels of associated gene expression. Recently, the existence of this cyclic ER binding to chromatin was called into question (177). However, the method used by Holding et al relies on using the insulator protein CTCF to normalize the ER chromatin profile, with multiple studies showing interconnectivity between these 2 proteins (57, 178-180), thereby potentially confounding the resolution of the normalization approach. In addition, it is unclear whether the lack of cycling observed by Holding et al is a phenomenon linked to the specific method used (ie, ChIP-seq might lack the resolution required to observe cycling) or a more general conclusion.

Understanding whether ER binding is cyclical or sustained could reveal the mechanism involved in shuttling ER off chromatin, a mechanism which could be exploited to prevent transcription of ER-regulated genes driving cell proliferation such as MYC or CCND1. Furthermore, FOXA1 binding is not cyclical, suggesting this shuttling mechanism could be ER specific. It would also be interesting to test whether cycling is observed for any of the ER-activating mutants (ie, Y537S/D538G). As these variants are characterized by a differential interactome compared with wild-type ER (96, 181), this might indicate whether different interaction partners contribute to cyclical ER binding. Together, work to define the mechanisms which underpin ER nuclear mobility and its stability on chromatin may ultimately provide an alternative strategy of manipulating ER activity.

## Future Perspectives

Despite significant progress in our understanding of the factors involved in the ER signaling pathway and their role in enhancer functionality in breast cancer cells, it is important to consider that, experimentally, most assays were performed on a population of cells. This represents a pooled measure and therefore likely neglects the gene/locus-specific dynamics of the ER-associated enhancer regulation. Implementation of CRISPR-Cas9 based approaches to target and annotate single functional enhancer elements (180, 182, 183) and to study the dynamics of locus-specific interactome using dCAS9–APEX-mediated proximity labeling (184) can provide novel opportunities to define the underlying mechanism in high resolution and in a gene-specific manner. In addition, detailed subcomplex investigations are required, in order to understand how the multitude of proteins work together, what cofactors are physically associated and what cofactors might be mutually exclusive. At the moment, common ER-associated cofactors appear to be in the same complex, but it is highly likely that numerous distinct subcomplexes exist, with ER as the common variable. Methodological approaches are currently being developed to address these issues, with the goal of deconvoluting the different subcomplexes. Our understanding of what cofactors are important, what mutations in specific cofactors should be studied and how changes (ie, deletion or copy number gains) of specific cofactors impact ER transcriptional activity and protein assembly on the chromatin, requires an understanding of the cofactor subcomplexes. Information on the cofactor subcomplexes will permit a deeper investigation into the potential dynamics in chromatin binding, complex reorganization, and cofactor degradation and displacement.

Furthermore, in vivo evidence of how some of these key regulators control enhancers and mediate disease progression is still lacking, which renders it difficult to assess their clinical relevance. One of the greatest challenges for future epigenetic studies remains obtaining fresh/frozen matched primary and metastatic tumor material. This requirement is necessary for understanding the evolution of drug resistance or the development of metastasis, both scenarios representing the poorest outcome in HR+ patients. The ability to carry out chromatin occupancy analyses on primary tumor material and biopsies (19, 185) and the recent technical advancement in the quantitative detection of endogenous protein interactions from patient samples (83) represent promising approaches to advance research in the in vivo setting. Additional developments using Assay for Transposase-accessible chromatin (ATAC-seq) (186, 187), CUT&RUN and CUT&TAG methods (188, 189) may facilitate our understanding of enhancer control in patient samples, where starting material is limited. As these techniques develop and become viable for use in single cells, it will be possible to dissect intratumor heterogeneity and the evolutionary metastatic trajectories of individual enhancer elements. In addition, the development of better, more physiologically relevant models of ER+ breast cancer invasion and metastasis such as those generated using the mouse mammary intraductal (MIND) approach will permit the investigation of key proteins and complexes involved in the metastatic process (190-192).

These technical advancements need to be accompanied by changes in our appreciation of how to tackle chromatin regulatory factors therapeutically. The majority of epigenetic players that are altered in cancer show context-dependent functions and can either limit or enhance cell growth depending on the tissue (influenced by the driving TF and the stoichiometry of associated cofactors in that tissue), disease stage (early versus advanced disease) or dose (partial or complete loss of activity) (193-195). While this context-dependent outcome underlines the inherent plasticity of the epigenomic environment, it is more difficult to integrate into our established criteria for classical drug targets. Novel approaches may also be required to improve the therapeutic index of such epigenetic drugs, which inevitably is affected when targeting proteins with multiple, complex, and potentially tissue-dependent antagonistic functions. However, dissecting the dynamic interplay between proteins with opposing functions and determining which epigenetic alterations are causative as opposed to correlative for different cancers can reveal a new layer of gene regulation at which therapy might be directed.

## Data Availability

No original data are provided in this manuscript.

## Abbreviations

ARID1A: AT rich interactive domain 1A
CHD4: chromodomain helicase DNA-binding protein 4
ChIP-seq: chromatin immunoprecipitation-sequencing
CRISPR: clustered regularly interspaced short palindromic repeats
CTCF: CCCTC-binding factor
ER: estrogen receptor alpha
eRNA: enhancer RNA
FOXA1: Forkhead Box A1
HDAC: histone deacetylase
HR+: hormone receptor positive
NuRD: nucleosome remodeling and deacetylase
PR: progesterone receptor
PRC: Polycomb repressive complex
SERD: selective estrogen receptor degrader
SERM: selective estrogen receptor modulator
TF: transcription factor
TRPS1: trichorhinophalangeal syndrome type I

## Additional Information

***Disclosures:*** The authors have nothing to disclose.
